# Terpenoids from the Marine-Derived Fungus *Aspergillus* *fumigatus* YK-7

**DOI:** 10.3390/molecules21010031

**Published:** 2015-12-28

**Authors:** Yu Wang, Da-Hong Li, Zhan-Lin Li, Yan-Jun Sun, Hui-Ming Hua, Tao Liu, Jiao Bai

**Affiliations:** 1 Department of Natural Products Chemistry, School of Pharmacy, China Medical University, Shenyang 110122, China; xiaowangyu21@163.com; 2 Key Laboratory of Structure-Based Drug Design & Discovery, Ministry of Education, Shenyang Pharmaceutical University, Shenyang 110016, China; lidahong0203@163.com (D.-H.L.); lzl1030@hotmail.com (Z.-L.L.); huimhua@163.com (H.-M.H.); 3 School of Traditional Chinese Materia Medica, Shenyang Pharmaceutical University, Shenyang 110016, China; 4 School of Pharmacy, Henan University of Traditional Chinese Medicine, Zhengzhou 450046, China; sunyanjun2011@hactcm.edu.cn

**Keywords:** marine-derived fungus, *Aspergillusfumigatus*, terpenoid, cell growth inhibition

## Abstract

Two new β-bergamotane sesquiterpenoids, *E*-β-*trans*-5,8,11-trihydroxybergamot-9-ene (**1**) and β-*trans*-2β,5,15-trihydroxybergamot-10-ene (**2**), were isolated from the marine-derived fungus *Aspergillus* *fumigatus* YK-7, along with three known terpenoids **3**–**5**. Their structures were determined by spectroscopic methods (1D and 2D NMR, HR-ESI-MS). Antiproliferative effects on human leukemic monocyte lymphoma U937 and human prostate cancer PC-3 cell lines were measured *in vitro*. Compound **4** exhibited potent activity against the U937 cell line with an IC_50_ value of 4.2 μM.

## 1. Introduction

**Figure 1:**
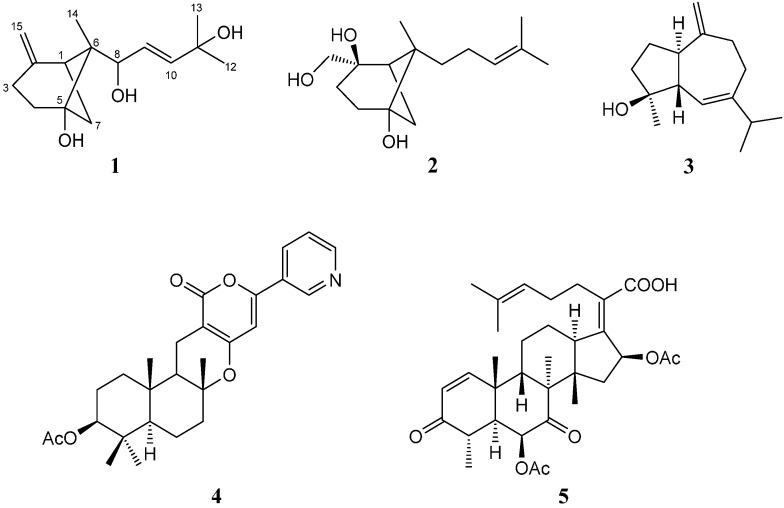
Structures of compounds **1**–**5**.

Microbial secondary metabolites are an important source of lead compounds for new drug development [1,2]. *Aspergillus* *fumigatus* has been found to generate many structurally and biologically diversified metabolites [3]. Among them, fumagillin is a meroterpenoid, and its derivatives have been studied for their potential use in the treatment of microsporidiosis [4] and amebiasis [5], and for their antiangiogenic properties exemplified by the irreversible inhibition of human type 2 methionine aminopeptidase (MetAP2) [6]. In our search for novel antitumor compounds from marine microorganisms, an extract of the fungus *Aspergillus* *fumigatus* YK-7, which was isolated from the sea mud of intertidal zone collected from Yingkou, China, exhibited significant activity against the U937 human leukemic monocyte lymphoma cell line (IC_50_ < 6.25 μg/mL). Previous investigation of this fungus had led to the isolation of fourteen 2,5-diketopiperazines [7]. In the course of our ongoing study on this fungus, two new β-bergamotane sesquiterpenoids **1** and **2** having a rare skeleton among fungal-derived metabolites and three known terpenoids **3**–**5** (Figure 1) were isolated from its fermentation broth. Compounds **1** and **2** may be the important intermediates in the biosynthesis of fumagillin and its derivatives [8]. Details of the isolation, structure elucidation, and cell growth inhibitory activities of these metabolites against U937 human leukemic monocyte lymphoma and PC-3 human prostate cancer cell lines are described here.

## 2. Results and Discussion

**Figure 2:**
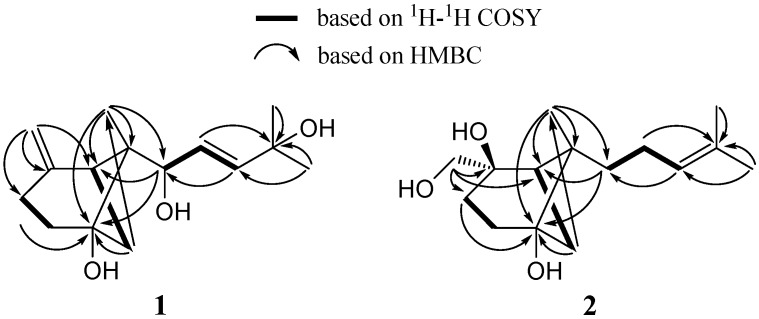
Key ^1^H-^1^H COSY and HMBC correlations of compounds **1** and **2**.

Compound **1** was obtained as a colorless oil. The molecular formula was demonstrated to be C_15_H_24_O_3_, indicating four degrees of unsaturation, based on HR-ESI-MS (*m*/*z* 275.1587 [M + Na]^+^; calc. 275.1623) in combination with NMR data. The ^13^C-NMR spectrum showed 15 carbon signals. Analyses of the ^1^H-, ^13^C-NMR, and HSQC spectra of **1** (Table 1) revealed the presence of three tertiary methyls (δ_H_ 0.80, δ_C_ 10.9; δ_H_ 1.33, δ_C_ 29.8; δ_H_ 1.33, δ_C_ 30.1), terminal (δ_H_ 4.57 and 4.67, δ_C_ 108.0; δ_C_ 147.9) and 1, 2-disubstituted (δ_H_ 5.66, δ_C_ 125.4; δ_H_ 5.88, δ_C_ 140.5) double bonds, an oxymethine (δ_H_ 4.85, δ_C_ 74.5), and two oxygenated quaternary carbons (δ_C_ 70.9; 76.7). These spectroscopic features together with the molecular formula indicated that **1** was a sesquiterpenoid. Since two double bonds accounted for two of the four degrees of unsaturation, **1** was concluded to be bicyclic. The HMBC spectrum of **1** (Figure 2) showed that the exomethylene protons H-15 (δ_H_ 4.57 and 4.67) were correlated with C-1 (δ_C_ 42.2), C-2 (δ_C_ 147.9), and C-3 (δ_C_ 25.3), and H-3 (δ_H_ 2.32 and 2.61) and H-7 (δ_H_ 1.91) were correlated with the oxygenated carbon C-5 (δ_C_ 76.7). These results, as well as the COSY correlations (Figure 2) of H-1 (δ_H_ 2.33) with H-7 (δ_H_ 1.91 and 2.47) and of H-3 (δ_H_ 2.32 and 2.61) with H-4 (δ_H_ 1.79 and 1.98), indicated the presence of a 4-methylene cyclohexanol ring. Specifically, the HMBC correlations of H-1 (δ_H_ 2.33), H-7 (δ_H_ 1.91), and H-4 (δ_H_ 1.79) with C-6 (δ_C_ 52.5), and of CH_3_-14 (δ_H_ 0.80) with C-1 (δ_C_ 42.2), C-5 (δ_C_ 76.7), and C-6 (δ_C_ 52.5), led to the assignment of a 6-methylbicyclo[3.1.1]heptane skeleton. Additionally, the COSY correlations from H-8 to H-10, and the HMBC correlations of the olefinic proton H-9 (δ_H_ 5.66) with the oxygenated carbon C-11 (δ_C_ 70.9), and of the olefinic proton H-10 (δ_H_ 5.88) with C-12 (δ_C_ 29.8) and C-13 (δ_C_ 30.1) suggested the presence of a 1,4-dihydroxy-4-methylpent-2-enyl side chain in **1**. The linkage of the two moieties was secured by the HMBC correlations of H-8 (δ_H_ 4.85) and H-9 (δ_H_ 5.66) with C-6 (δ_C_ 52.5). Therefore, compound **1** was established as a β-5,8,11-trihydroxybergamot-9-ene [9,10].

**Figure 3:**
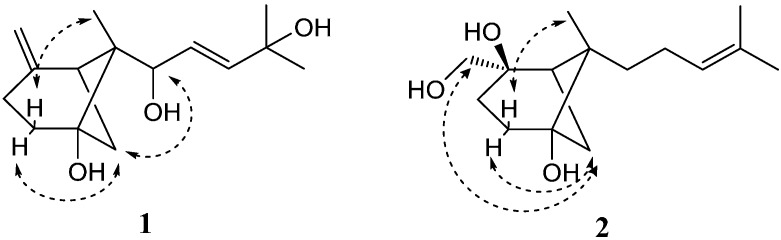
Key NOESY correlations of compounds **1** and **2**.

The geometry of the Δ^9^ double bond was assigned as *E* on the basis of a coupling constant 15.7 Hz (*J*_H-9,10_). The 6-methyl-endo configuration was determined by NOESY correlations (Figure 3) of H-4β (δ_H_ 1.98) with CH_3_-14 (δ_H_ 0.80), H-7 (δ_H_ 1.91) with H-4α (δ_H_ 1.79), and of H-7 (δ_H_ 2.47) with H-8 (δ_H_ 4.85), and was further confirmed by the comparison of ^1^H-NMR chemical shift data for CH_3_-14 (δ_H_ 0.80) with the literature values (δ_H_ 0.71 for β-*trans*-bergamotene; δ_H_ 1.23 for β-*cis*-bergamotene) [11]. Thus, the structure of **1** was assigned as *E*-β-*trans*-5,8,11-trihydroxybergamot-9-ene, although the absolute configuration was not defined.

Compound **2** was obtained as colorless needles. The molecular formula C_15_H_26_O_3_ from HR-ESI-MS (*m*/*z* 277.1737 [M + Na]^+^; calc. 277.1780) indicated that it possessed two more hydrogen atoms than compound **1**. The ^1^H- and ^13^C-NMR data of **2** (Table 1) showed similarity to those of **1**, suggesting the presence of another β-bergamotane skeleton. However, a hydroxymethyl group (δ_H_ 3.34 and 3.47; δ_C_ 69.4) linked to the oxygenated carbon C-2 (δ_C_ 76.5) in **2** replaced the exomethylene group in **1**, which was confirmed by the HMBC correlations (Figure 2) of H-15 (δ_H_ 3.34 and 3.47) with C-1 (δ_C_ 39.7), C-2 (δ_C_ 76.5), and C-3 (δ_C_ 29.4). Moreover, the side chain in **2** was different from that in **1**, which was established as 4-methylpent-3-enyl by the COSY correlations (Figure 2) from H-8 to H-10, and the HMBC correlations of H-9 (δ_H_ 2.03 and 2.10) with C-11 (δ_C_ 131.8), and of the olefinic proton H-10 (δ_H_ 5.15) with C-12 (δ_C_ 17.8) and C-13 (δ_C_ 25.8). NOESY correlations (Figure 3 of H-4β (δ_H_ 2.05)/CH_3_-14 (δ_H_ 1.18), H-4α (δ_H_ 1.75)/H-7 (δ_H_ 1.47), and H-7 (δ_H_ 1.47)/H-15 (δ_H_ 3.34 and 3.47) indicated the 6-methyl-*endo* configuration and the α-orientation of the hydroxymethyl group. Consequently, the structure of **2** was defined as β-*trans*-2β,5,15-trihydroxybergamot-10-ene. The known compounds alismol (**3**) [12], pyripyropene E (**4**) [13], and helvolic acid (**5**) [14,15], were identified by comparison of their spectroscopic data with those reported in the literature.

**Table 1:** ^1^H- and ^13^C-NMR data for compounds **1** and **2** in CDCl_3_.

	1		2	
Position	δ_C_^a^	δ_H_^b^ (*J* in Hz)	δ_C_^a^	δ_H_^b^ (*J* in Hz)
1	42.2	2.33, d (7.5)	39.7	2.15, m
2	147.9		76.5	
3	25.3	2.32, 2.61, m	29.4	1.82, 1.86, m
4	31.5	1.79 (α), 1.98 (β), m	30.8	1.75 (α), 2.05 (β), m
5	76.7		76.2	
6	52.5		46.9	
7	36.1	1.91, d (10.0)	36.1	1.47, 2.17, m
		2.47, dd (10.0, 7.5)		
8	74.5	4.85, d (6.1)	34.5	1.45, 1.70, m
9	125.4	5.66, dd (15.7, 6.1)	23.2	2.03, 2.10, m
10	140.5	5.88, d (15.7)	124.9	5.15, t (7.1)
11	70.9		131.8	
12	29.8	1.33, s	17.8	1.62, s
13	30.1	1.33, s	25.8	1.68, s
14	10.9	0.80, s	17.8	1.18, s
15	108.0	4.57, br. s	69.4	3.34, d (10.8)
		4.67, br. s		3.47, d (10.8)

^a^ Recorded at 75 MHz; ^b^ Recorded at 300 MHz.

All compounds were evaluated *in vitro* for cell growth inhibitory activities against the U937 and PC-3 cell lines (IC_50_ values are shown in Table 2). Compound **4** exhibited potent selective inhibition against U937 cell line, with the IC_50_ value of 4.2 μM, and **1**, **3**, and **5** exhibited weak activities against U937 cell line with IC_50_ values of 84.9, 61.7, and 57.5 μM, respectively. All the compounds didn’t show antiproliferative effect in PC-3 cell lines.

**Table 2:** Antiproliferative activity (IC_50_ (μM)) of compounds **1**–**5** on U937 and PC-3 cells ^a^.

Compound	U937 Cells	PC-3 Cells
**1**	84.9 ± 2.4	>100
**2**	>100	>100
**3**	67.1 ± 1.9	>100
**4**	4.2 ± 0.3	>100
**5**	57.5 ± 3.2	>100
Doxorubicin hydrochloride	0.021 ± 0.002	0.73 ± 0.04

^a^ U937 cells were treated for 3 days, and PC-3 cells were treated for 4 days. IC_50_ value is the concentration that inhibited 50% of cell growth. The data shown are means ± S.D. of three independent experiments.

## 3. Experimental Section

### 3.1. General Procedures

Optical rotations were obtained on a Perkin-Elmer 241MC polarimeter (Perkin-Elmer, Waltham, MA, USA). The IR spectra were recorded on Bruker IFS-55 spectrometer (Bruker Optics, Ettlingen, Germany). NMR spectra were recorded on Bruker ARX-300 or AV-600 NMR spectrometers (Bruker Biospin, Fallanden, Switzerland), with TMS as the internal standard. HR-ESI-MS was performed on a Bruker microTOF-Q mass spectrometer (Bruker Daltonics, Billerica, MA, USA). Chromatographic silica gel (200–300 mesh) was purchased from Qingdao Marine Chemical Factory (Qingdao, China), and ODS (50 μm) was obtained from YMC Co. Ltd. (Kyoto, Japan). The RP-HPLC analysis and semi-preparation were conducted using a Hitachi L2130 series pumping system (Hitachi, Tokyo, Japan) equipped with a Hitachi L2400 UV detector (Hitachi, Tokyo, Japan) and a YMC-PACK ODS-AM column (250 × 10 mm, 5 μm, YMC, Kyoto, Japan). TLC spots were visualized under UV light (Zhengzhou Greatwall Scientific Industrial and Trade Co., Ltd., Zhengzhou, China) and with 10% H_2_SO_4_ in EtOH followed by heating.

### 3.2. Fungal Material

The fungus, YK-7, was isolated from an intertidal zone sea mud sample collected from Yingkou, China, and identified as *Aspergillus fumigatus* by its morphological characteristics and ITS sequences [7,16]. A voucher strain was deposited at −80 °C in School of Traditional Chinese Materia Medica, Shenyang Pharmaceutical University (Shenyang, China).

### 3.3. Extraction and Isolation

The fungus was cultivated at 28 °C for 7 days while shaking at 165 rpm in 300 500 mL flasks containing a liquid medium (150 mL per flask) composed of 3 g of yeast extract, 1 g of corn steep liquor, 20 g of mannitol, 10 g of monosodium glutamate, 10 g of glucose, 20 g of maltose, 0.5 g of KH_2_PO_4_, and 0.3 g of MgSO_4_·7H_2_O, per 1000 mL seawater at pH 6.5.

The fermented whole broth (45 L) was filtered through a cheesecloth into the supernatant and the mycelia. The supernatant was concentrated under reduced pressure to about 5 L, partitioned with EtOAc (3 × 5 L) at room temperature, and then dried by rotary evaporation to yield a crude extract (21 g), which showed significant growth inhibitory activity against the U937 cell line (IC_50_ < 6.25 μg/mL). The crude extract was subjected to column chromatography (CC) (SiO_2_; CHCl_3_/MeOH gradient) to yield 17 fractions, Fr. 1–17. Fr. 2 (100:1) was purified by repeated CC (SiO_2_; petroleum ether (PE)/acetone 100:15; and ODS; MeOH/H_2_O 65:35) to afford **5** (15 mg). Fr. 3 (100:2) was subjected to repeated CC (SiO_2_; PE/acetone 100:25; and ODS; MeOH/H_2_O 90:10) to afford **3** (21 mg). Fr. 5 (100:5) was fractionated by CC (ODS; MeOH/H_2_O) to give seven subfractions, subfrs. 5-1–5-7. Subfr. 5-3 (40:60) was purified by CC (SiO_2_; PE/acetone 2:1) to afford **2** (3 mg). Subfr. 5-4 (50:50) was further subjected to CC (SiO_2_; PE/acetone 4.5:1) to yield **1** (3 mg). The mycelia were extracted with acetone (3 × 3 L) at room temperature and then dried by rotary evaporation. The crude extract (250 g; IC_50_ < 6.25 μg/mL) was subjected to CC (SiO_2_; CHCl_3_/MeOH gradient) to yield 14 fractions, Fr. 1–14. Fr. 2 (100:1) was separated by CC (SiO_2_; PE/acetone gradient) to give six subfractions, subfrs. 2-1–2-6. Subfr. 2-4 (5:1) was further purified by semipreparative HPLC (MeOH/H_2_O 85:15; t_R_ = 38 min) to afford **4** (6 mg).

*E-β-trans-5,8,11-trihydroxybergamot-9-ene* (**1**): colorless oil; ${\left[\alpha \right]}_{D}^{22}$ −21.6 (*c* 0.11, MeOH); IR(KBr) ν_max_ 3426, 2920, 2851, 1643, 1460, 1384, 1129, 879 cm^−1^; ^1^H- and ^13^C-NMR data, see Table 1; HR-ESI-MS *m*/*z* 275.1587 [M + Na]^+^ (Calcd for C_15_H_24_O_3_Na, 275.1623). The IR, NMR, and HR-MS spectra of compound **1** can be found at Supplementary Material (Figures S1–S8).

β*-trans-2β,5,15-trihydroxybergamot-10-ene* (**2**): colorless needles (MeOH); mp 115–116 °C; ${\left[\alpha \right]}_{D}^{22}$ −18.5 (*c* 0.10, MeOH); IR(KBr) ν_max_ 3405, 2921, 2852, 1642, 1452, 1383, 1150, 1052, 954 cm^−1^; ^1^H- and ^13^C-NMR data, see Table 1; HR-ESI-MS *m*/*z* 277.1737 [M + Na]^+^ (Calcd for C_15_H_26_O_3_Na, 277.1780). The IR, NMR, and HR-MS spectra of compound **2** can be found at Supplementary Material (Figures S9–S16).

### 3.4. Cell Culture and Growth-Inhibition Assay

The growth inhibitory assay was performed as described previously [17,18]. Human leukemic monocyte lymphoma U937 and human prostate cancer PC-3 cell lines (American Type Culture Collection, Rockville, MD, USA) were cultured in RPMI-1640 medium (Gibco, New York, NY, USA) supplemented with 100 U/mL penicillin, 100 μg/mL streptomycin, 1 mmol glutamine, and 10% heat-inactivated fetal bovine serum. The growth-inhibitory ability of these crude extracts and isolated compounds was calculated and expressed as the ratio of the cell number in treated group to that of the untreated group. The concentration that inhibited half of the cell growth, IC_50_, was calculated. Doxorubicin hydrochloride (Hua Bo Technology Co. Ltd., Beijing, China) was used as a positive control, and 0.1% DMSO was used as a negative control.

## Supplementary Materials

The IR, NMR, and HR-MS spectra of compounds **1** and **2** are available online at: http://www.mdpi.com/1420-3049/21/1/31/s1.
