# Supplementary material for: Terpenoids from the Marine-Derived Fungus Aspergillus fumigatus YK-7
Source: Molecules. 2015 Dec 28;21(1):31. doi: 10.3390/molecules21010031 (PMC6273775; doi:10.3390/molecules21010031)
Supplement: Supplementary file 1 [file molecules-21-00031-s001.pdf]

Supplementary Materials: Terpenoids from the Marine-Derived Fungus *Aspergillus fumigatus* YK-7

Yu Wang [1], Da-Hong Li [2,3], Zhan-Lin Li [2,3], Yan-Jun Sun [4], Hui-Ming Hua [2,3], Tao Liu [1,*] and Jiao Bai [2,3,*]

Figure S1. HR-ESI-MS spectrum of compound **1**.
Figure S2. ^{1}H-NMR (300 MHz, $CDCl_3$) of compound **1**.
Figure S3. ^{13}C-NMR (75 MHz, $CDCl_3$) of compound **1**.
Figure S4. ^{1}H-^{1}H COSY (600 MHz, $CDCl_3$) of compound **1**.
Figure S5. HSQC (600 MHz, $CDCl_3$) of compound**1**.
Figure S6. HMBC (600 MHz, $CDCl_3$) of compound **1**.
Figure S7. NOESY (600 MHz, $CDCl_3$) of compound **1**.
Figure S8. IR spectrum of compound **1**.
Figure S9. HR-ESI-MS spectrum of compound **2**.
Figure S10. ^{1}H-NMR (300 MHz, $CDCl_3$) of compound **2**.
Figure S11. ^{13}C-NMR (75 MHz, $CDCl_3$) of compound **2**.
Figure S12. ^{1}H-^{1}H COSY (600 MHz, $CDCl_3$) of compound **2**.
Figure S13. HSQC (600 MHz, $CDCl_3$) of compound **2**.
Figure S14. HMBC (600 MHz, $CDCl_3$) of compound **2**.
Figure S15. NOESY (600 MHz, $CDCl_3$) of compound **2**.
Figure S16. IR spectrum of compound **2**.

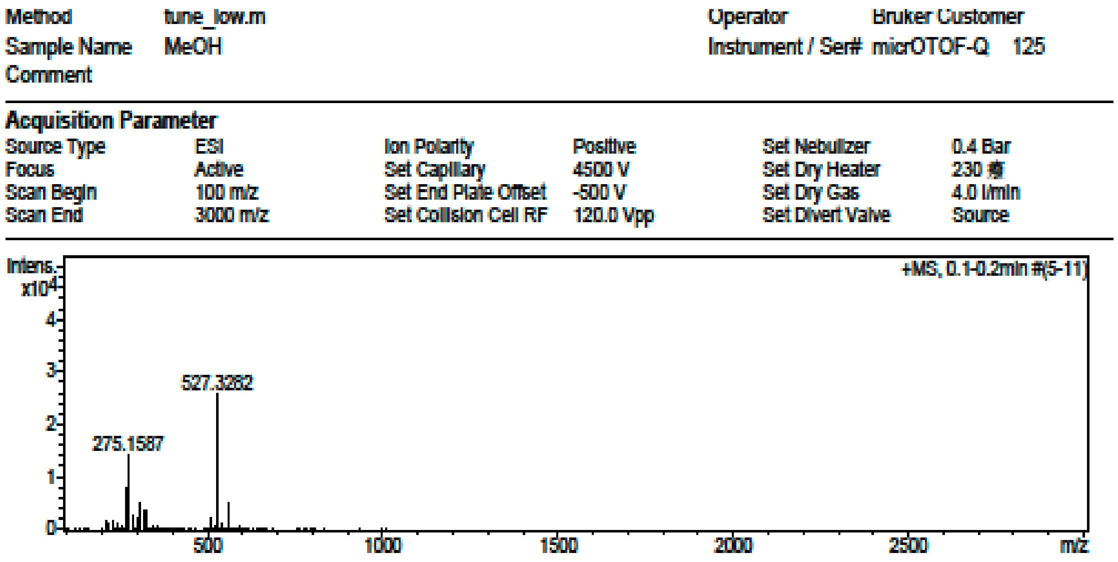

Figure S1. HR-ESI-MS spectrum of compound **1**.

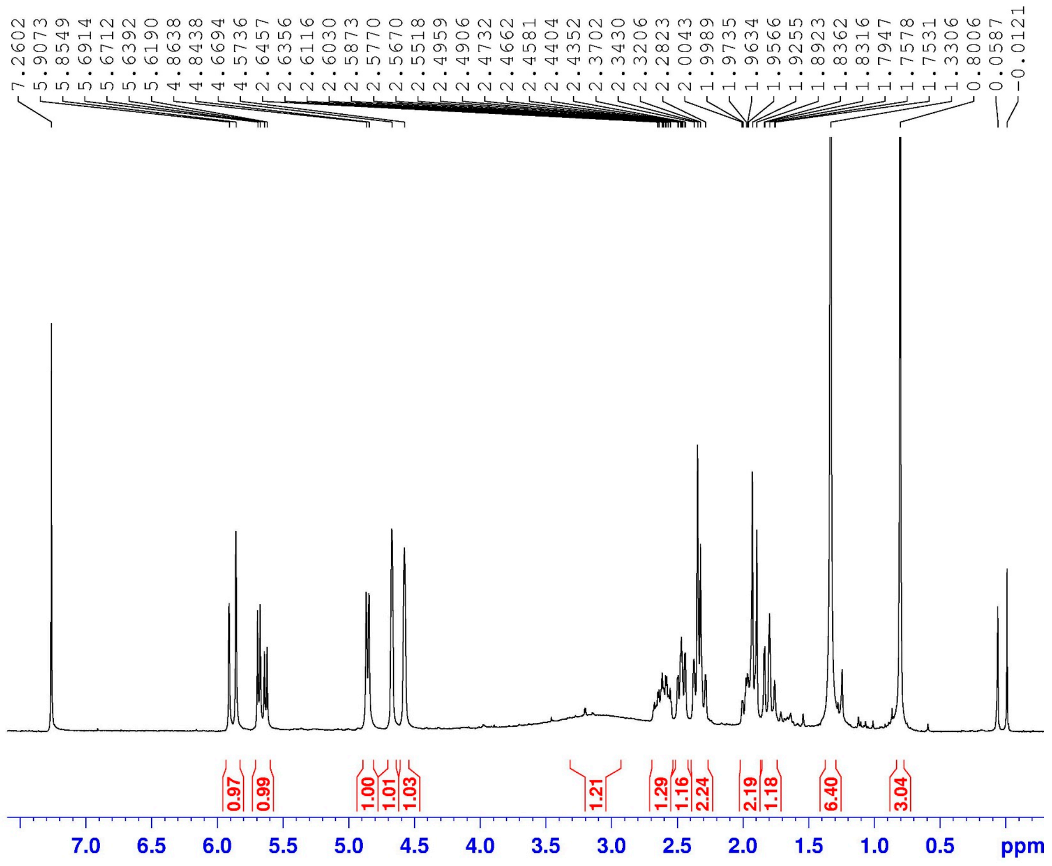

Figure S2. ^{1}H-NMR (300 MHz, $CDCl_3$) of compound **1**.

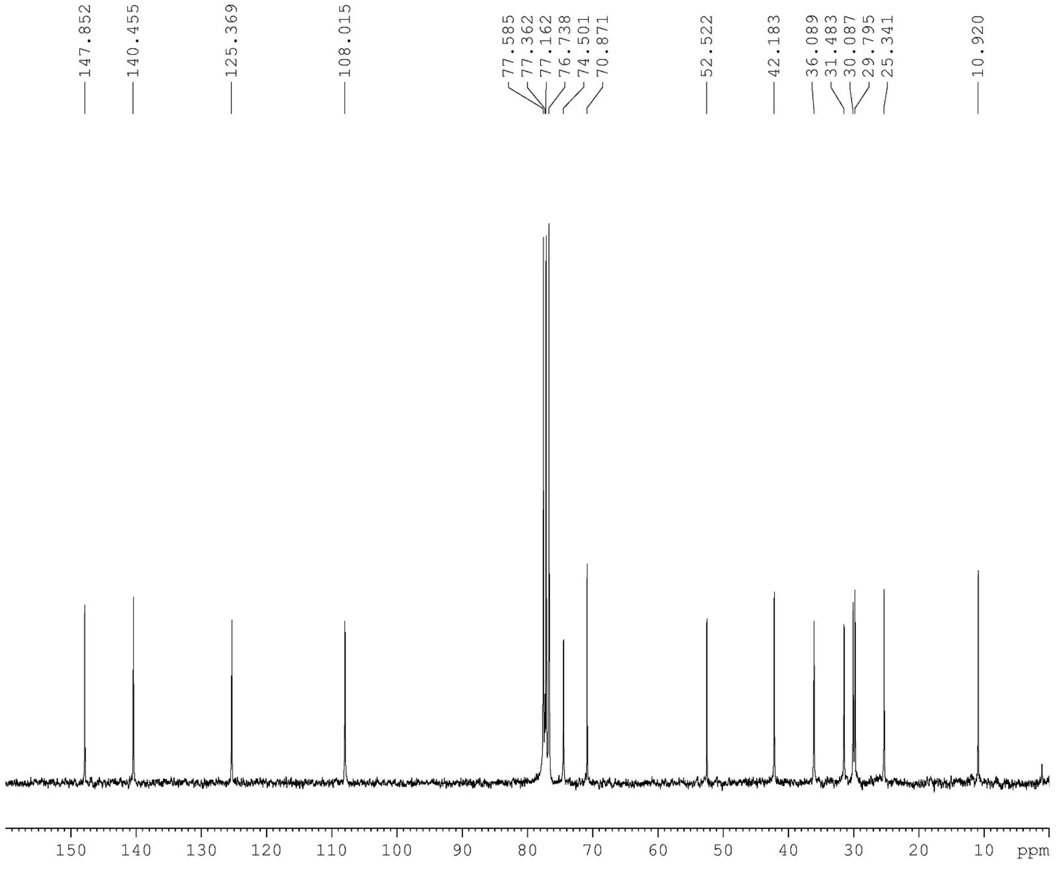

Figure S3. ^{13}C-NMR (75 MHz, $CDCl_3$) of compound **1**.

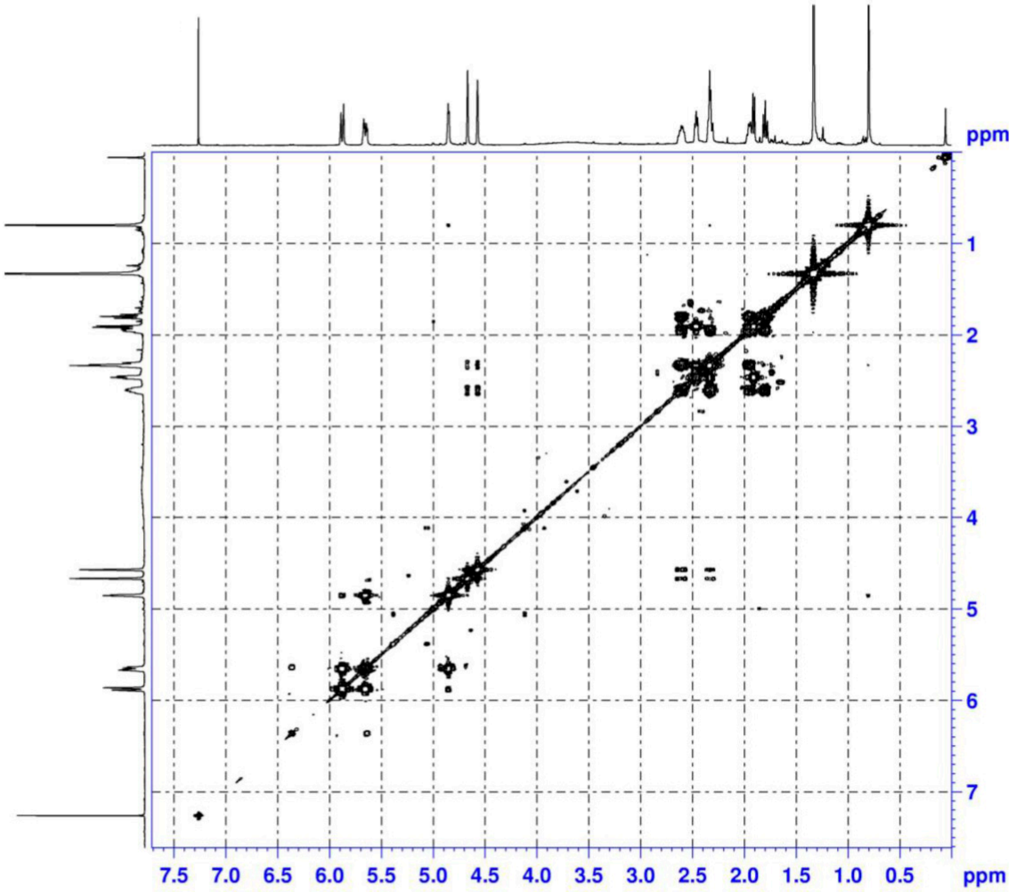

Figure S4. ^{1}H-^{1}H COSY (600 MHz, $CDCl_3$) of compound **1**.

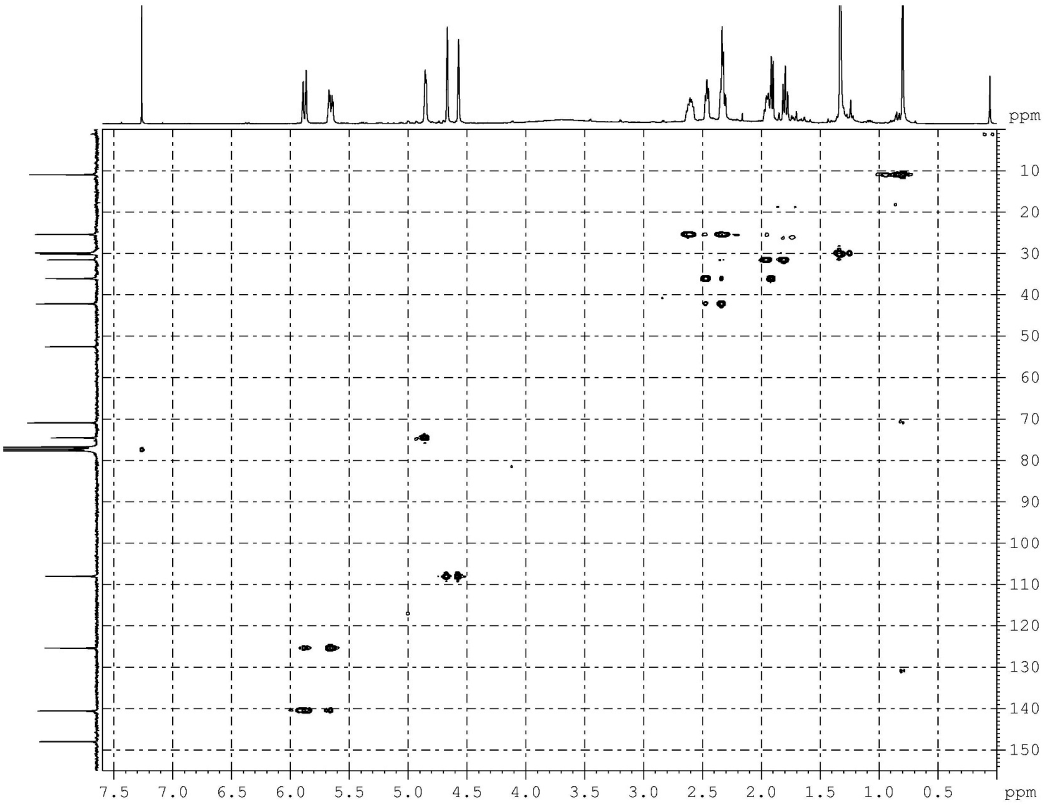

Figure S5. HSQC (600 MHz, $CDCl_3$) of compound **1**.

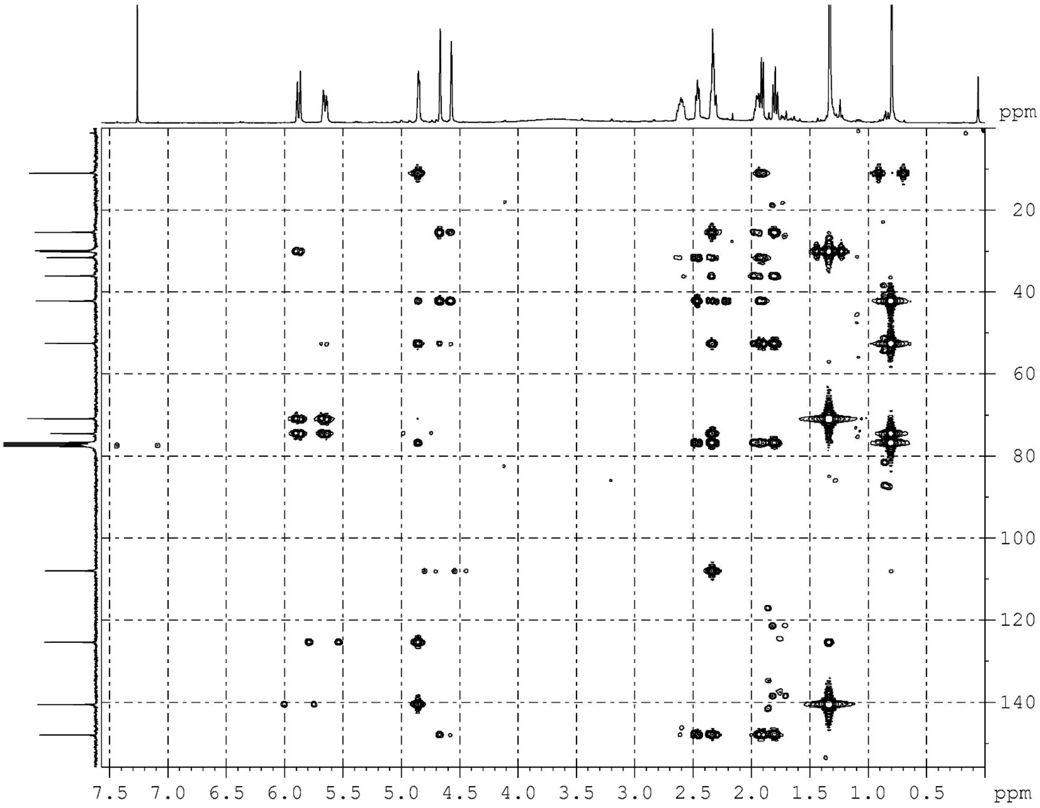

Figure S6. HMBC (600 MHz, CDCl3) of compound **1**.

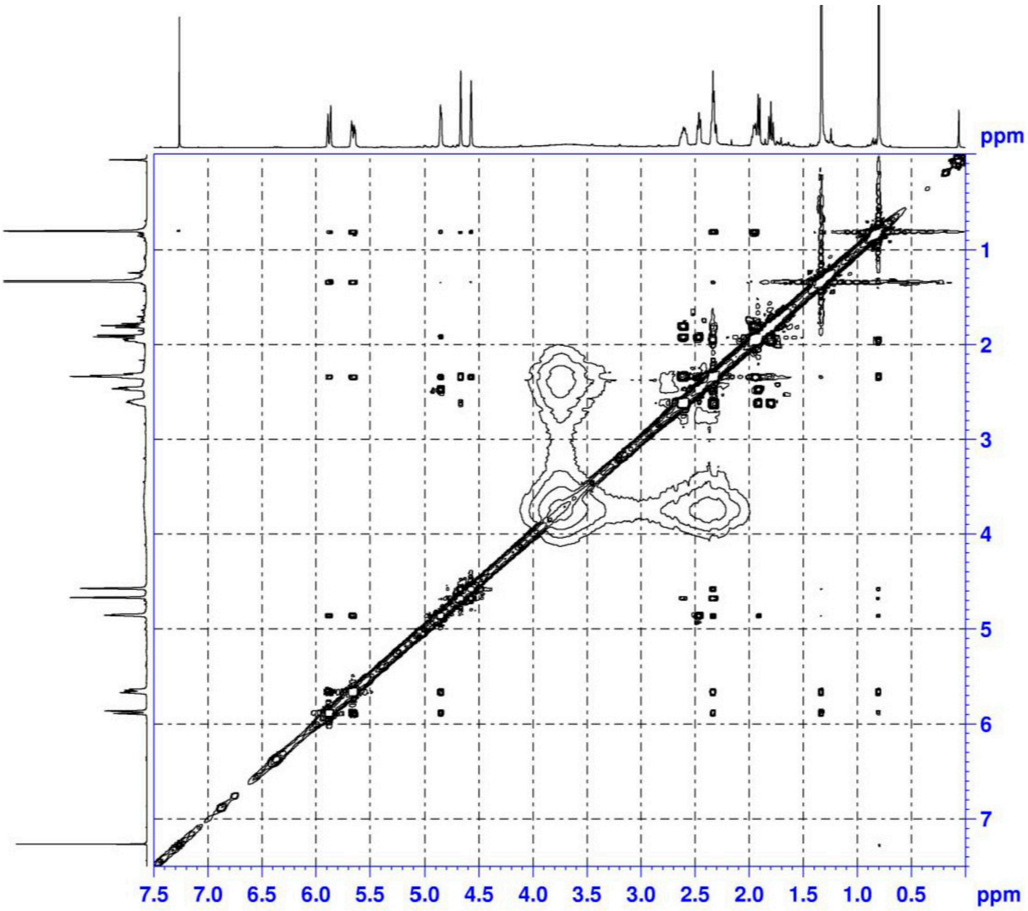

Figure S7. NOESY (600 MHz, CDCl3) of compound **1**.

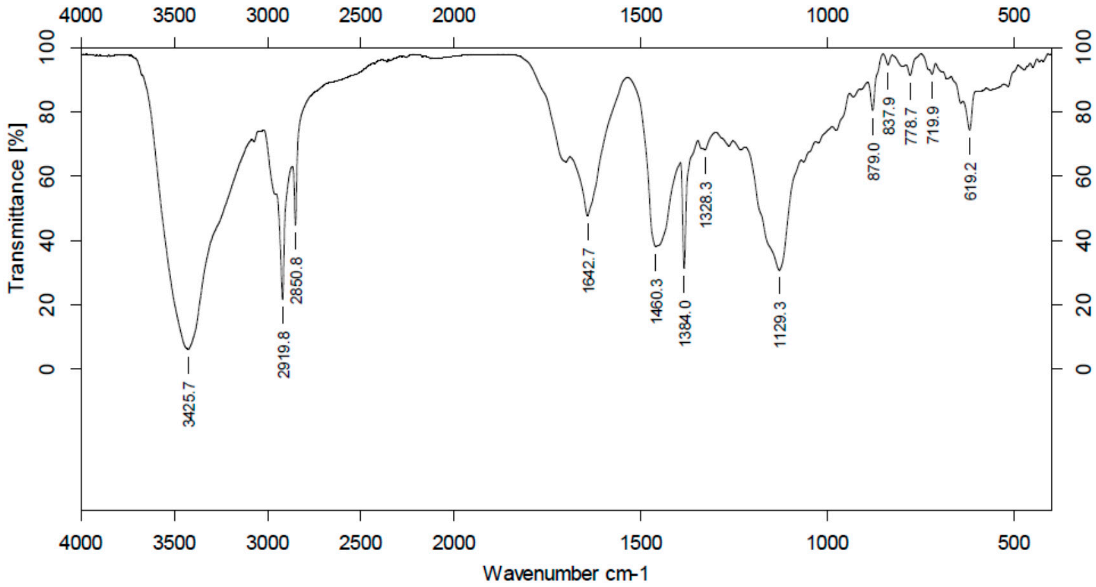

Figure S8. IR spectrum of compound **1**.

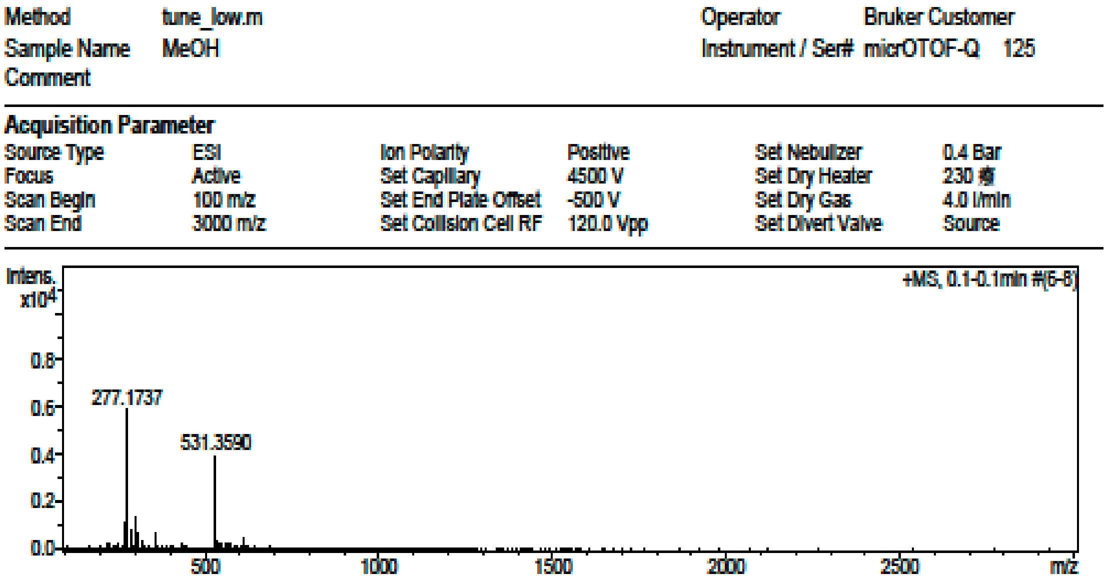

Figure S9. HR-ESI-MS spectrum of compound **2**.

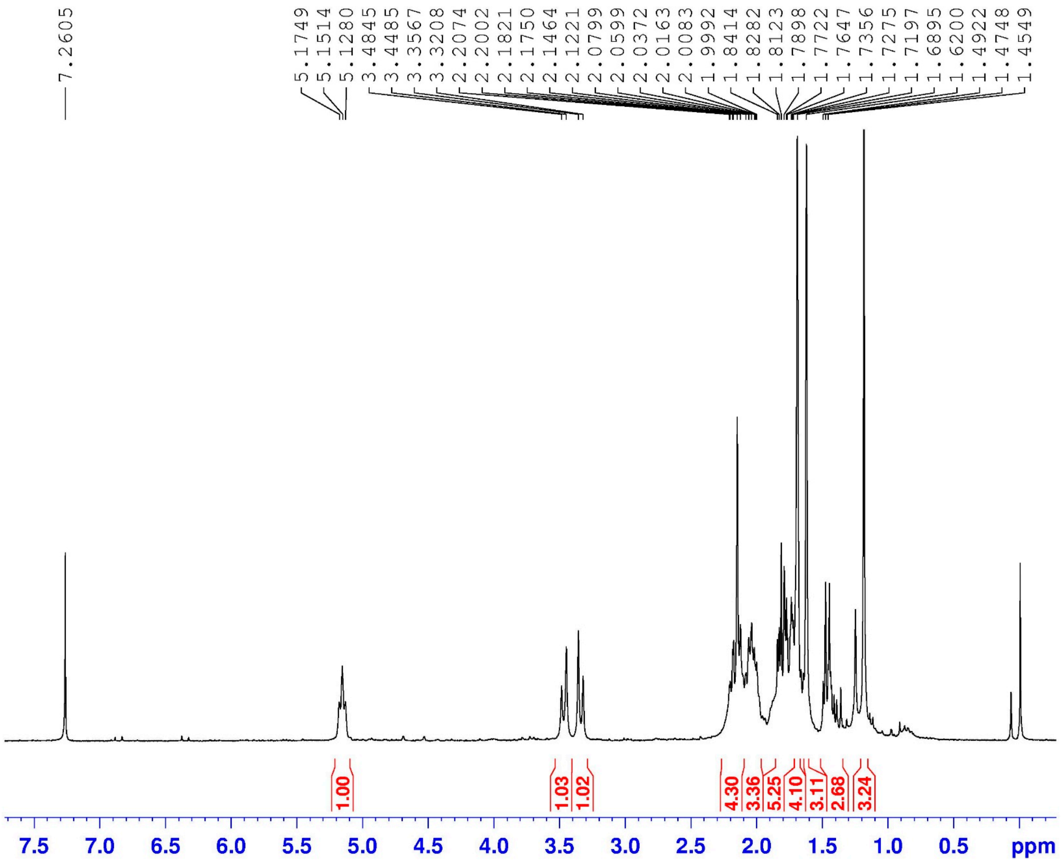

Figure S10. ^{1}H NMR (300 MHz, $CDCl_3$) of compound **2**.

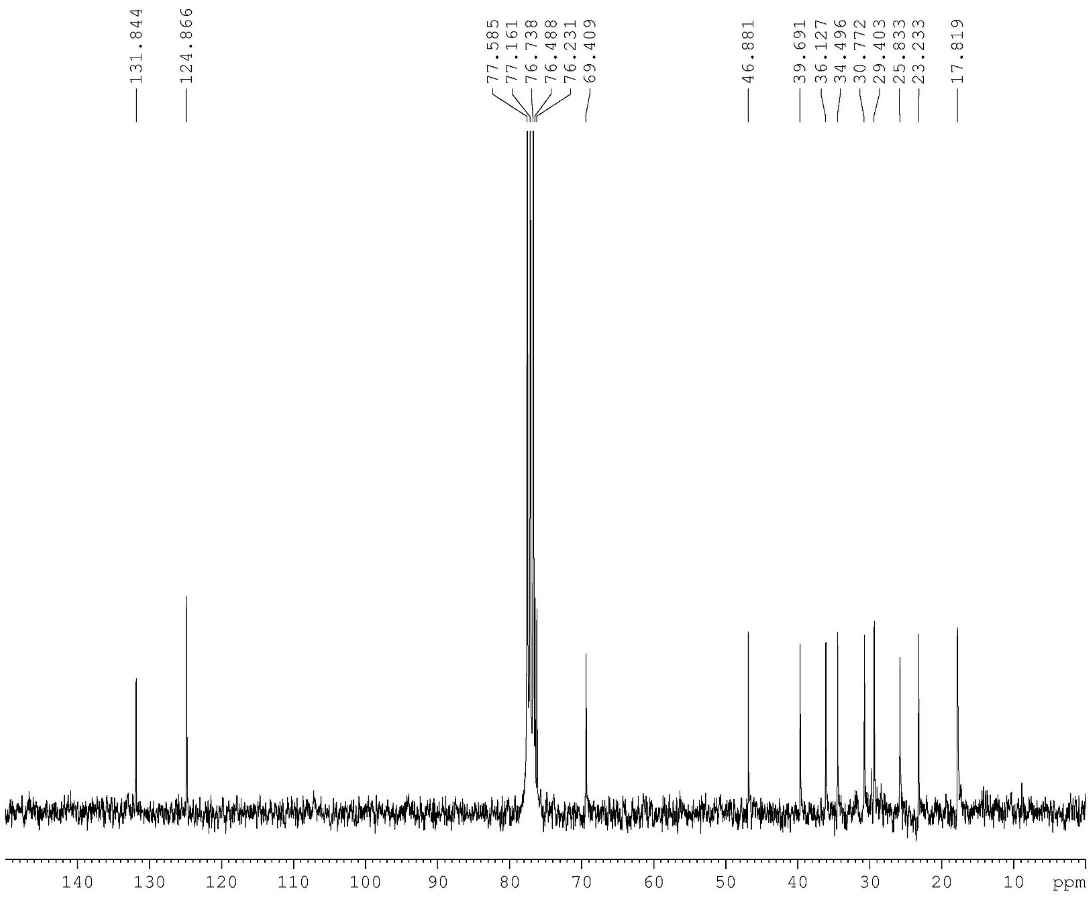

Figure S11. ^{13}C-NMR (75 MHz, $CDCl_3$) of compound **2**.

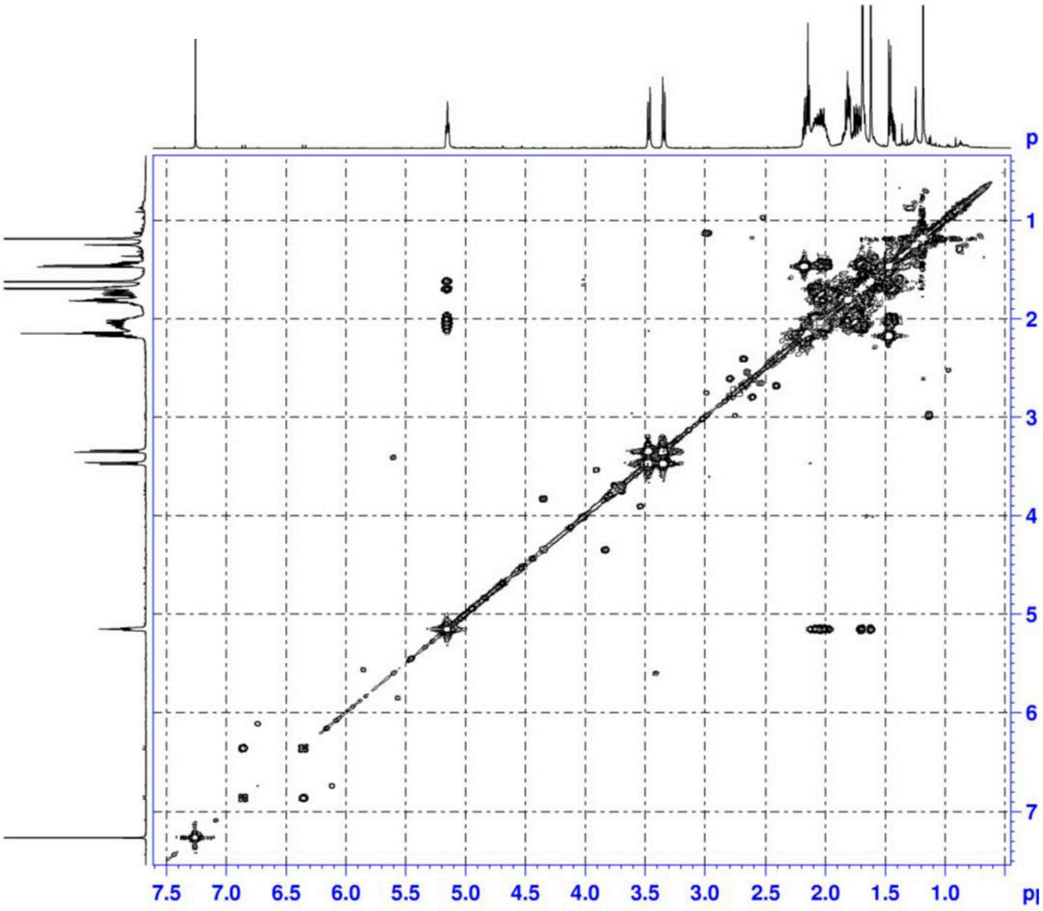

Figure S12. ^{1}H-^{1}H COSY (600 MHz, $CDCl_3$) of compound **2**.

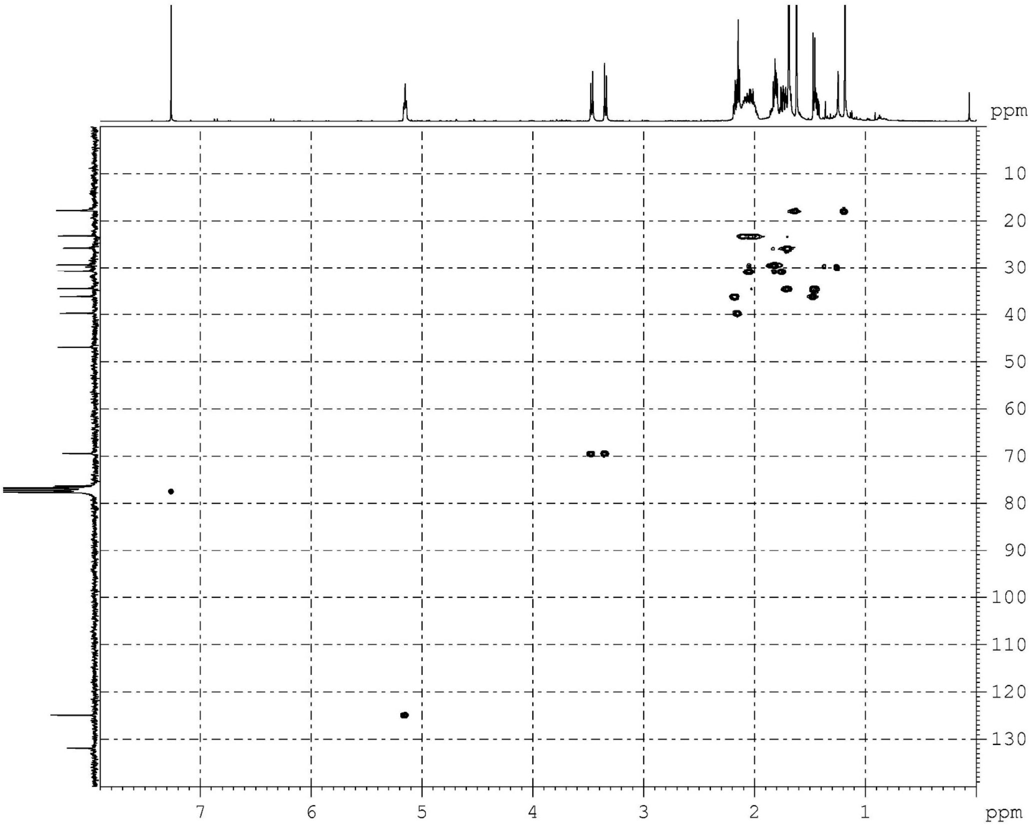

Figure S13. HSQC (600 MHz, $CDCl_3$) of compound **2**.

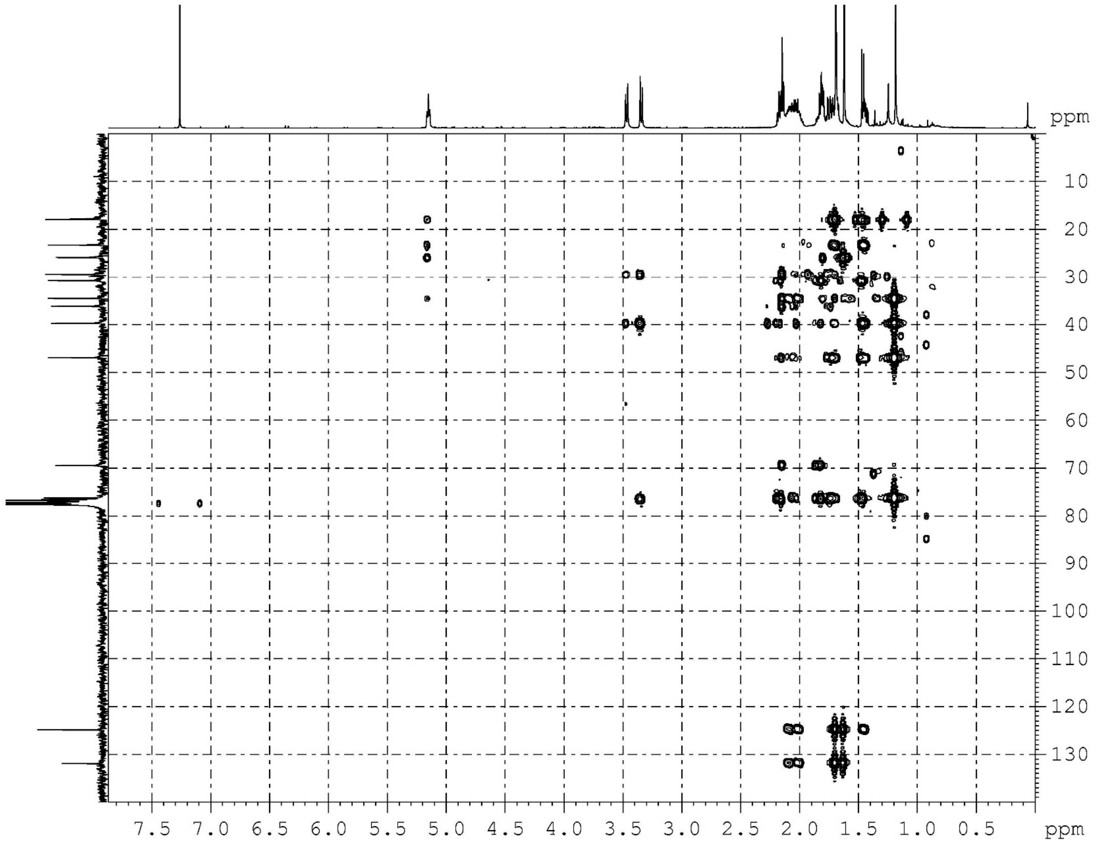

Figure S14. HMBC (600 MHz, $CDCl_3$) of compound **2**.

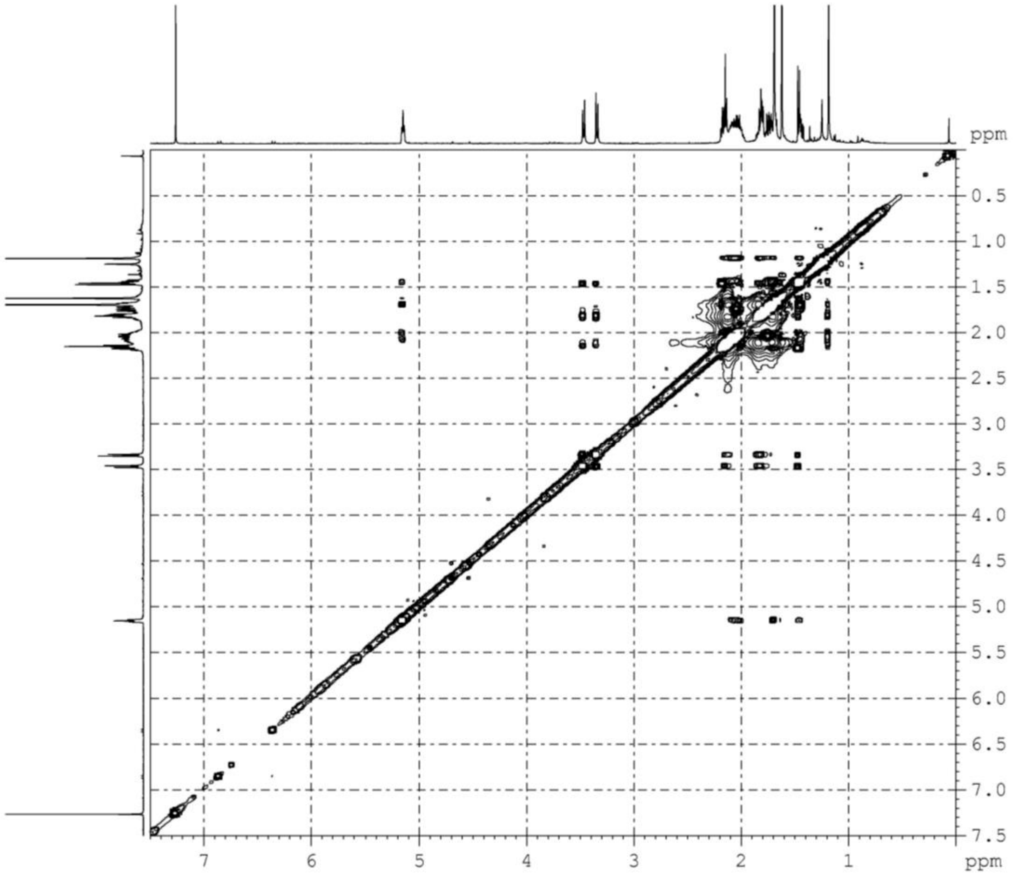

Figure S15. NOESY (600 MHz, $CDCl_3$) of compound **2**.

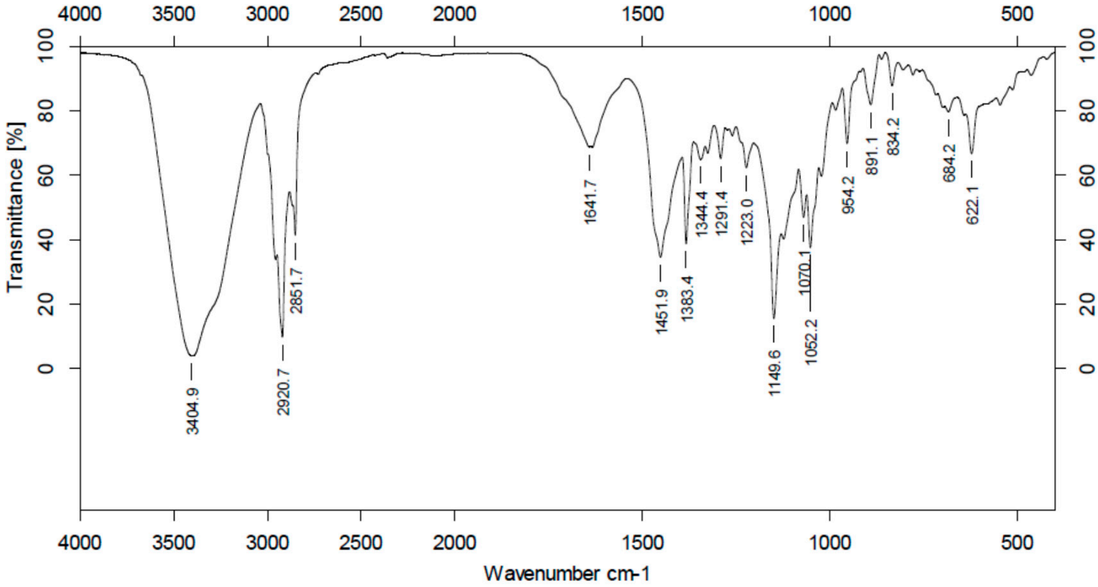

Figure S16. IR spectrum of compound **2**.
